# Lawsuit lead time prediction: Comparison of data mining techniques based on categorical response variable

**DOI:** 10.1371/journal.pone.0198122

**Published:** 2018-06-01

**Authors:** Lúcia Adriana dos Santos Gruginskie, Guilherme Luís Roehe Vaccaro

**Affiliations:** 1 Graduate Program in Production Engineering and Systems, Unisinos, São Leopoldo, Rio Grande do Sul, Brazil; 2 Graduate Program in Business and Management, Unisinos, Porto Alegre, Rio Grande do Sul, Brazil; Northwestern University, UNITED STATES

## Abstract

The quality of the judicial system of a country can be verified by the overall length time of lawsuits, or the lead time. When the lead time is excessive, a country’s economy can be affected, leading to the adoption of measures such as the creation of the Saturn Center in Europe. Although there are performance indicators to measure the lead time of lawsuits, the analysis and the fit of prediction models are still underdeveloped themes in the literature. To contribute to this subject, this article compares different prediction models according to their accuracy, sensitivity, specificity, precision, and F1 measure. The database used was from TRF4—the Tribunal Regional Federal da 4^a^ Região—a federal court in southern Brazil, corresponding to the 2^*nd*^ Instance civil lawsuits completed in 2016. The models were fitted using support vector machine, naive Bayes, random forests, and neural network approaches with categorical predictor variables. The lead time of the 2^*nd*^ Instance judgment was selected as the response variable measured in days and categorized in bands. The comparison among the models showed that the support vector machine and random forest approaches produced measurements that were superior to those of the other models. The evaluation of the models was made using k-fold cross-validation similar to that applied to the test models.

## Introduction

The quality of a judicial system can be verified by the quality of judgments, the efficient use of public resources, the access to justice for all citizens, and the overall case length [1]. The effects of unsatisfactory performance of the judiciary have repercussions on the economy of any country as they discourage commercial transactions, add risks and costs, and reduce the size of the market and its competitiveness [2].

One example of poor performance is the excessive length of court proceedings. Time affects the judicial system in many ways: in the civil system, negative effects include the costs of waiting for compensation, the deterioration of evidence, and the decrease in the time allocated to scrutinizing settlements by judges, all motivated by an interest in reducing the workload [3]. The perception of the sluggishness of the judiciary motivates the inclusion of its reformation in the economic and social development agendas of institutions such as the World Bank and the Council of Europe [4]. Article 6 of the European Convention on Human Rights determines that courts must settle their cases within a reasonable time, though no clear definition of ‘reasonable’ is suggested. The concern with the overall case length has spurred CEPEJ—The European Commission for the Efficiency of Justice—to set up a working group to manage procedural time called the Saturn Center, which has a network of test courts with members from different countries [5].

In Brazil, the Amendment 45 to the Brazilian Constitution from 2004 imposes a reasonable length of lawsuits and the means that ensure the speed of its proceedings. To control and manage this time, it is necessary to measure, analyze and predict. Measurement of lawsuit time is performed in both Brazil and Europe; however, the overall case length prediction models represent a developing theme in the academy. Some studies aim to model lead time through regression analysis, for example, [6, 7], and in the Brazilian literature, there are examples of the use of artificial neural networks (NNs) [8].

Approaches attempting to solve this problem use the regression analysis technique to predict the duration of the civil lawsuit [6], to predict the length of time to reach a resolution to a criminal case [9], and to analyze the factors that influence this timing [10, 11]. Usually, the response variable is quantitative, and the independent variable is binary or quantitative [6, 9–11]. Other approaches using text mining [12–15] and sentiment analysis [16] also may be found for other types of applications in the judicial system.

Considering the context presented above, this article aims to fit and compare models for predicting the overall case length in the Brazilian court system with a focus on civil lawsuits in the federal courts of Second Instance. Data from TRF4 with a one-year timespan are used to support the analysis. Recently, TRF4 has become famous as the court that is responsible for ‘lava jato’ recursal actions and has been mentioned in international journals such as the New York Times.

In contrast to the articles mentioned above, the dependent variable is categorized into classes by time, and the categorical variables are used as independent variables. To model the data, support vector machine (SVM), naive Bayes (NB), random forest (RF) and NN approaches are used. A comparison of performance is given in terms of accuracy, sensitivity, specificity, precision, and F1 measures.

This paper is segmented into five sections, including this introduction. The next section presents the background on measuring the overall case length in the judiciary system, related works, and the approaches and variables used in these studies. Next, the material and methods section addresses the references on the SVM, RF, NB, and NN approaches in addition to evaluations and parameters for fitting these models. Finally, the conclusion section presents suggestions for future papers.

## Background

First, an overview of the concepts and systems employed in the measurement of overall case length is provided. Next, a review of the works related to the theme published in the EBSCO database and the Brazilian works is provided.

### Measurement of lead time

To understand the duration of a lawsuit, to identify the reasons for delays and to predict the duration of a lawsuit, it is necessary to measure this duration. Monitoring the duration of judicial proceedings should be performed through an integrated and well-defined system for the collection of information [5]. This system should provide detailed data on the duration of the process and general and per-instance characteristics and should define an excessive period in advance. Specifically, in relation to measuring the duration of lawsuits, several different terms and indicators for measurement are used as well as formal indicators in different systems. The following is a brief summary of indicators relating to lead time.

The Budget Law Project from the French Judicial Justice program has produced indicators to define *ex ante* objectives and assess *ex post* performances [17].

The Global Measures of Court Performance describe eleven measures aligned with the values and areas of court excellence, answering the questions “What to measure” and “How to measure it” [18]. These measures are in accordance with universally accepted court values and are identified by the International Framework for Court Excellence (IFCE). Among the eleven measures is on-time case processing, or the percentage of cases resolved within the established timeframe.

To measure the performance of courts in a manageable way, there are 10 CourTools system indicators, including disposition time, or the percentage of cases eliminated or solved within the timeframe [19]. In the European Court of Justice, the CPMS—Court Performance Measure System, which is defined by the international laboratory for the study of judicial systems, was based on the balanced scorecard and includes the following five dimensions [20]: client satisfaction, internal operation, financial characteristics, innovation and learning, and the success of information systems. Disposition time is among the indicators of internal operation.

In Brazil, the Conselho Nacional de Justiça (CNJ), through the Justice in Numbers Report, publishes the following indicators regarding procedure time [21] as measured using mean, median and standard deviation:

- Overall lead time;
- Lead time of unresolved cases;
- Lead time for trials;
- Lead time of interruption.

In these systems of indicators, time is measured through several indexes with different marks for the beginning and end of the lawsuit. The main concepts sought in the literature in reference to the judicial sphere presented in this article as indicators of time are disposition time and court delays.

The disposition time is the number of days between the arraignment date and the case disposition date [9], such as the date of a plea negotiation or a trial outcome. However, this indicator is also defined as the number of days required for a pending case to be resolved, [20]. On practice, 365 is divided by the indicator case turnover ratio, or the ratio of cases settled to unsettled cases. This last definition for the indicator does not take into account the duration of the lawsuit.

In criminal lawsuits, the time for which a case remains in the judicial system includes the actual processing time, i.e., the time that a judge, clerks and prosecutors dedicate to a particular case; and waiting times [3], which is similar to the lead time concept in production engineering. Lead time can be defined as the time taken to transform raw materials into products or services, and this concept can be applied broadly (lead time of the client) when the purpose is to measure the time from the request until the actual delivery or in a restricted way (production lead time) in order to measure the internal activities of the production system [22]. In the judicial context, lead time can be interpreted as the time between arraignment in the judicial system and case disposition, and according to the circumstances, these events may be considered simultaneous both from the point of view of the user and the production system.

There are criticisms and observations about time in the literature. In the judicial context, delay is a relative concept because there is no clear definition of “too slow” [10, 23]. If in such a global assessment, the court finds that the total time is excessive or that there were long periods of inactivity by authorities, the reasonable time can be said to have been exceeded. For this declaration, the cause of the delay should be known [5].

The overall case length, or lead time, is an important measure of court efficiency, and although this variable alone cannot adequately capture information on satisfaction with the court system, it is a component of satisfaction and is considered very important [7].

The reasonable duration of a lawsuit is not precisely defined [24], so the times and the characteristics of the lawsuits (such as the demands, complexities and specifics) should have receive attention in the analysis.

In this article, the time of procedural duration will be used as the lead time in the 2^*nd*^ Instance, that is, the time from the arraignment to the final disposition but without the intermediate times and measured in time bands.

### Related works

In Brazil, the concept of jurimetrics has been applied in academic works in recent years. Jurimetrics, a term presented by Loevinger in 1949, is defined as the quantitative analysis of judicial behavior, the application of communication and information theory to legal expression, the use of mathematical logic in law, the retrieval of legal data by electronic and mechanical means and the formulation of a calculation of legal predictability [25].

To find quantitative studies, a search was made in the EBSCO database in March 2017 without filters. The keywords length of judicial proceedings, court delay, disposition time, filings court time and time to court case resolution were searched to identify the variable lead time. The keywords data mining, regression analysis, survival analysis, support vector machine, random forest and naive Bayes were searched to identify quantitative analyses. The results are presented in Table 1. Regression analysis was the technique most commonly used in lawsuit lead time analysis.

**Table 1:** Related works on overall case length.

Keyword	Support Vector Machine	Naive Bayes	Random Forests	Data Mining	Regression Analysis	Survival Analysis
Length of judicial proceedings	0	0	0	2	4	0
Court delay	0	0	0	2	19	3
Disposition time and court	0	0	0	1	51	2
filings court time	0	0	0	0	0	0
Time to Court Case Resolution	0	0	0	0	0	0

Source: The authors

Using the above key words, 11 articles related to this study were found and are presented here. These articles present models to predict the duration of civil lawsuits [6, 7] and the factors that influence this timing [3, 10, 11, 26]. Other articles address the duration of criminal cases [9, 27], while others describe methodologies and techniques [28–30]. The following is a brief description of the objectives, data and analysis techniques used in these works.

- A regression model to predict the duration of the process based on the data from the Texas Department of Insurance Commercial Liability Insurance Closed Claim Report for the period 1988-2005. The two main reasons for the delays are found to be when one of the litigants has an interest in the delay and when the delay is caused by congestion in court [6].
- A model for predicting the overall case length of civil cases using from the IAALS (Advancement of the American Legal System at the University of Denver) study. The authors used a database of civil cases terminated from October 1^*st*^, 2005 to September 30^*th*^, 2006, with approximately 6700 cases in 7 federal districts [7].
- An analysis of the factors that determine the duration of a case with a focus on two particular subjects, namely, medical negligence and personal injury, using linear regression with a transformed dependent variable. [11].
- A study of the factors that affect the length of criminal judicial proceedings, how this delay affects social welfare and how the prosecutor’s incentives induce him to press or refrain from prosecution. The developed model uses queuing theory to represent the way that congestion in the courts is created, making it is possible for decision makers to effectively compare different policy proposals and examine their effects on lead time. The authors also addressed the prosecutor’s incentives for a set of defendants when the criminal justice system is clogged [3].
- The construction of a conceptual framework based on the literature to categorize the determinants of lead time at three levels, namely, country, court and cases, emphasizing the first and third levels. This is the first article to analyze the time of civil lawsuits in the Belgian First Instance courts through an ordinary least squares approach. The author used a database of 174 cases, and the indicators were selected based on the existing literature. Additional indicators were identified after discussions with a panel of legal and academic experts, focusing on cases that appeal to the chamber judging construction and settlement disputes (construction cases) and the chamber adjudicating tax disputes [26].
- A proposal for a methodology describing an economic model of criminal courts, applied to the analysis of the following three issues: i) costs and benefits related to criminal cases by trial or fault, ii) the ideal total number of judgments in a criminal court, and iii) the optimal allocation of criminal lawsuits according to the type of infractions within a certain judicial capacity [28].
- A study on the practical operation of the reasonable time protection approach described in article 6 of the convention, taking particular account of the Italian case and assessing the benefits and problems associated with this solution. The Pinto Act involved an unproven interaction between the Italian Supreme Court (’Corte di Cassazione’) and the ECtHR, promoting a new debate on the effects of ECtHR jurisprudence on the Italian legal system [29].
- The development of hypotheses and methodologies to explain the determinants of the length of civil lawsuits. This work presents an explanatory tool to structure future empirical investigations and lays the foundations for the creation of robust time standards by which civil courts can be measured more accurately and monitored and compared across jurisdictions. The authors proposed a common vocabulary and an empirical methodology for comparative studies of the problem of lead time in civil justice. The authors used DEA—data envelopment analysis—to compare the courts and used regression analysis to explain the causes of differences in efficiency. [10].
- The development of a theoretical model with explicit criteria for explanatory variables for time, which is considered as the number of days between the arrest warrant (presentation of the defendant in the court) and dismissal, judgment or appeal. The authors confront the question in a mathematical way, making the model nonlinear and non-additive, and use a two-year sample of Detroit court cases. The analysis is based on a random sample of crimes/complaints [30].
- A study to understand how courts resolve child sexual abuse crimes by addressing the time needed to reach a criminal case resolution. The study analyzes how time period varies with the crime using a regression analysis. The other objectives of this study were to explore whether child sexual abuse offenses differed with the use of the resolution of measure 11 and to assess the perceptions of the main legal stakeholders (lawyers, judges and court administrators) on local legal culture and the criminal resolution of child sexual abuse crimes [9].
- A lead time analysis of criminal cases using machine learning techniques to forecast the time that defendant will take to go through the criminal justice system from the date of arrest to the date of the termination of the case using the following process: imprisonment, police disposition, provision of the prosecutor/grand jury, final judicial disposition, and sentence. Another objective of the article was to determine which factors have the greatest influence on lead time [27].

In addition to these articles, using technical and scientific research approaches, works in the Brazilian justice system were researched with the same parameters used on the EBSCO website. Before the brief description of the works found, the organization of the Brazilian judiciary is presented.

The Brazilian judiciary is composed of a) the Federal Supreme Court (STF), b) the National Council of Justice (CNJ), c) the Superior Court of Justice (STJ) d) the Common Justice court, and e) the Specialized Justice court [31]. The STF is the highest level of the Brazilian judiciary and comprises the functions of the Supreme Court and the constitutional court. Below the STF is the STJ, which is responsible for a uniform interpretation of federal legislation.

The Specialized Justice court is formed by the electoral, labor and military courts, each with the respective competences. The Common Justice court is composed of federal courts and the Courts of States. The federal courts are responsible for the cases in which the Federal Government, its municipal organizations and federal public companies are represented as well as other issues of interest to the federation, while the Courts of States are responsible for judging issues that are not the jurisdiction of the federal court or any other specialized court. The works found refer to labor justice and the Courts of States and are described below:

- The identification of the relationships between time and several variables for lawsuits in the Court of the State of Rio Grande do Sul using cluster analysis and association rules based on municipal and public security data [32].
- The prediction of the duration of a lawsuit in a labor court from Paraná using cluster analyses and NNs [8].

These works from Brazil and other countries used several variables on lawsuit phases and the characteristics of the defendant, the plaintiff, the court and the action. The lawsuit phase variables are the time elapsed between when the case is filed and the initial scheduling conference, the number of motions brought under the Federal Rule of Civil Procedure and the elapsed time for the court to rule on each of these motions [7].

Regarding the profile of the defendant and plaintiff, the variables refer to the characteristics of the offender (age at the time of the incident, age at the time of referral to prosecution, race/ethnicity) [9, 27]. Regarding the author’s profile, the variables refer to the age at the time of the incident, age at the time of referral for prosecution and race / ethnicity) [9] and whether they are legal entities.

Regarding the characteristics of the court, the variables capture the judicial structures and resources, such as the number of judges, number of cases per judge (caseload), court salaries, number of buildings, and number of rooms, among others.

The main variables on the characteristics of the action in criminal justice are the judicial outcome (decline/dismiss, plea, acquittal, conviction at trial) [9]; the legal step in which the solution was reached (agreement by alternative dispute resolution mechanisms, settlement before trial, settlement before court verdict, court verdict, settlement after verdict, and trial or summary judgment) [6]; the legal complexity (the complexity of legal issues raised by the parties and the nature of the proceedings); factual/technical complexity (number of witnesses, number of experts, and number of expositions); litigation funding (partly funded, legal aid, or other external funds); dispute between corporations; dispute between individuals; dispute between a corporation and one or more individuals [10]; type of case; number of lawyers for the parties registered in the proceeding; holding of hearings; number of extensions requested or granted and extensions granted; history of appeals [7]; whether the defendant contributed to the amount paid; the level of severity of the complaint in levels [11]; expert involvement (binary); total number of allegations, total number of defendants; total number of plaintiffs; whether the plaintiff wins; and if the case is dependent on another process [26].

In relation to the intermediate times, the variables are the time elapsed between case filing and the initial scheduling conference, the elapsed time for the court to rule on each such motion, and the length and type of trial (with or without jury) [7]. The above works are important for contextualizing the variables and techniques already used in the literature to introduce the work conducted in this article.

For the comparison of performance methods, previous works have compared the performance of classifiers [33], including a comparison of the following nine classifiers implemented in the structure of Weka 9 for pattern recognition: NB, Bayes networks, RF, simple classification and regression tree (CART), C4.5, k-nearest neighbors (kNN), logistic regression, multilayer perceptron and support vector machine approaches.

## Materials and methods

First, this work presents a comparison of different approaches, the variables used to compose the models and an evaluation of the trained models.

The four predominant approaches used for the classification of lead time are SVM, NB, RF and NN. More details on these approaches can be obtained from [34–36].

The SVM approach was chosen because of its accuracy, the ability to model complex nonlinear decision boundaries, and a reduced tendency to overfitting compared to other methods [35]. NB was selected for its simplicity of application and satisfactory results, and this approach is appropriate when the feature space is high [34]. The RF approach was chosen for the ease of deriving the training fit [34], and artificial NNs were chosen because this approach has high noise tolerance and can be used when there is little knowledge of the relationships between attributes and classes [35].

### Support vector machine

SVMs have been applied to different areas such as handwriting, object and speaker recognition, benchmark time-series prediction tests [35], bioinformatics and astrophysics [37].

The training data consist of *N* pairs (*x*_1_, *y*_1_), (*x*_2_, *y*_2_), …, (*x*_*N*_, *y*_*N*_) with *x*_*i*_ ∈ *R*^*p*^ and *y*_*i*_ ∈ [−1, 1]. A hyperplane is defined as follows [34]:

$$\begin{array}{c} x:f\left(x\right)=x^{T}\beta +\beta_{0}=0 \end{array}$$ (1)

where *β* is a unit vector: ‖*β*‖ = 1.

Considering two classes, an SVM selects a particular hyperplane that separate the points into two classes and maximizes the margin, which is the distance between the hyperplane and the nearest points of the training set [38]. If each of these two parts has points only in this class, the dataset is said to be linearly separable [36] and can be formulated as follows [34]:

$$\begin{array}{c} Min_{\left(\beta ,\beta_{0}\right)∥\beta ∥} \end{array}$$ (2)

subject to $y_{i}\left(x_{i}^{T}\beta +\beta_{0}\right)\geq 1,i=1,...,N$. This relation is not considered the restriction of *β*, and $M=\frac{1}{∥\beta ∥}$.

SVM can handle non-separable points by introducing slack variables [36]. Slack variables, *ξ* = (*ξ*_1_, …, *ξ*_*N*_), are the values *ξ* = (*ξ*_1_, …, *ξ*_*N*_), *ξ* under the restriction $y_{i}\left(x_{i}^{T}\beta +\beta_{0}\right)\geq M\left(1-\xi_{i}\right)$, or the proportional amount by which the prediction $f\left(x_{i}\right)=x_{i}^{T}\beta +\beta_{0}$ is on the wrong side of its margin [34].

When delimiting the sum value of ∑*ξ*_*i*_, misclassification can occur when *ξ*_*i*_ ≥ 1, with the total number of these misclassification being equal to *K*. Then, *M* = 1/‖*β*‖. This relation is rewritten in the Lagrange form as follows:

$$\begin{array}{c} Min_{(\beta ,\beta_{0})}\frac{1}{2}{∥\beta ∥}^{2}+C\sum_{i=1}^{N}\xi_{i} \end{array}$$ (3)

subject to $\sum \xi_{i}\geq 0,y_{i}\left(x_{i}^{T}\beta +\beta_{0}\right)\geq 1-\sum \xi_{i},\forall i$ where *C* is the cost parameter [34]. When the goal is not to misclassify points and to accept a narrow margin, choose a large *C*. Choose a small *C* when the goal is to have most points located far from the limit (that is, with a large margin) but with some badly ranked points [38].

For the *m* classes extension, a simple approach is to train *m* classifiers, one for each class, where the *j* classifier returns a positive value for the class and a negative value for the other classes [35].

The base function *h*(*x*), *m* = 1, …, *M* fits a support vector classifier using *h*(*x*_*i*_) = (*h*_1_(*x*_*i*_), *h*_2_(*x*_*i*_), …, *h*_*M*_(*x*_*i*_)), *i* = 1, …, *N*, and then, the nonlinear function $\hat{f}\left(x\right)=h\left(x\right)T\hat{\beta }+\hat{\beta }0$ is produced [34]. The Lagrangian function has the following form:

$$\begin{array}{c} LD=\sum_{i=1}^{N}\alpha_{i}-\frac{1}{2}\left(\sum_{i=1}^{N}\sum_{i^{\prime }=1}^{N}\alpha_{i}\alpha_{i^{\prime }}y_{i}y_{i}^{\prime }\left\langle h\left(x_{i}\right)h\left(x_{i^{\prime }}\right)\right\rangle \right) \end{array}$$

The solution can be written as follows [34]:

$$\begin{array}{ccc} f(x) & = & h{\left(x\right)}^{T}\beta +\beta_{0}\\ f(x) & = & \sum_{i}^{N}\alpha_{i}y_{i}\left\langle h\left(x\right),h\left(x_{i}\right)\right\rangle +\beta_{0} \end{array}$$

Given *α*_*i*_, *β*_0_ can be determined by the solution to *y*_*i*_ *f*(*x*_*i*_) = 1 for 0 < *α*_*i*_ < *C*.

SVM uses an implicit mapping of input data defined by a kernel function, that is, a function returning the internal product between the images of two data points *x*, *x*′ in the feature space [37]. If a projection *ϕX* → *H* is used, the point product 〈*ϕ*(*x*)*ϕ*(*x*′)〉 can be represented by a kernel function as follows [37]:

$$\begin{array}{c} k\left(x,x^{\prime }\right)=\left\langle \phi \left(x\right)\phi \left(x^{\prime }\right)\right\rangle \end{array}$$ (4)

There are three functions of *K* [34] as follows:

- d^th^-degree polynomial: *K*(*x*, *x*′) = (1 + 〈*x*, *x*′〉)^*d*^,
- Radial basis: *K*(*x*, *x*′) = *exp*(−*γ*‖*x* − *x*′‖^2^),
- NN: *K*(*x*, *x*′) = *tanh*(*κ*_1_〈*x*, *x*′〉+*κ*_2_)

Then, the solution can be written as follows [34]:

$$\begin{array}{c} Min_{\left(\beta \beta_{0}\right)}\sum_{i}^{N}{\left[1-y_{i}f\left(x_{i}\right)\right]}_{+}\frac{\lambda }{2}{∥\beta ∥}^{2} \end{array}$$

For the choice of kernel function, [39] propose a multi-label learning-based kernel recommendation method with the objective of automatically selecting appropriate kernel functions for a given dataset. This method is based on the characteristics of the data, and there are references in [39]. The steps for an SVM Analysis are as follows [40]:

- Transform the data. Categorical data need to be transformed into a numerical format, and the authors suggest using *m* variables for an attribute with *m* categories. For each of these new variables, the value is 1 for the interest category and 0 for the others;
- Scale the data to fall within the interval [-1; 1] or [0; 1];
- Consider a kernel function radial basis function (RBF);
- Use cross-validation to find the best values of *C*, costs, and *γ*. In the kernel RBF function, there are two values to choose. The aim is to choose the best values to accurately predict the rating values in the test set;
- Use the best values of *C* and *γ* to train the dataset;
- Test.

### Naive bayes

The objective of the approach is to determine the probability that the tuple X belongs to class *C* once the description of the *X* attribute is known [35].

The computational simplicity of this approach is due to the assumption of independence, called conditional class independence [35]. The performance of the NB algorithm is comparable to that of decision trees and NNs because this approach has high accuracy and speed when applied to large databases [35] and offers fast training of the classifier and low computational complexity of *O*(*nd*) [36].

Although class density estimation can be biased, this bias may not prejudice the probabilities, especially near the decision regions [34]. For the test, the algorithm classifies a given point *x* by estimating the probability that the point belongs to each class, returning the class with the highest probability as a prediction, as follows [41]:

$$\begin{array}{c} p\left(c|E\right)=\frac{p\left(e_{1}|c\right)p\left(e_{2}|c\right)...p\left(e_{k}|c\right)p\left(c\right)}{p(E)} \end{array}$$

where *e*_*i*_ are the events and *c* is the class.

The probability *p*(*c*) can be estimated by counting the proportion of examples of class *c* among all examples, and *p*(*e*_*i*_|*c*) can be estimated by the proportion of points in class *c* for each characteristic because the violation of the assumption of independence between variables tends not to affect classification performance very much [41].

### Random forests

RFs are a modification of the bagging technique. RFs are popular and have been implemented in a series of computational packages [34]. This method is attractive for the following reasons [42]:

- There are no assumptions about data type or distribution, and the approach does not require prior processing;
- RF is a robust method for redundant and nonlinear data;
- The algorithm is easy to implement, and the output tree is relatively easy to understand;
- After fitting the model, attaining the score is fast.

However, RF does have some disadvantages [42]:

- There is a tendency for overfitting, especially without suppression;
- The training variance is high. Samples from the same population can produce trees with different structures and different prediction accuracies;
- The accuracy of the prediction may be low when compared to other methods;
- Usually, the bagging technique is used to improve models.

If each classifier in the ensemble is a decision tree classifier, then there is a forest where each tree depends on the values of a random vector sampled independently and that has the same distribution for all the trees in the forest [35]. For each individual tree in the set, the RF method follows the steps [34]:

- From the training data, a bootstrap sample is selected;
- For each sample and at each node of the tree, a decision tree grows;
- A subset of variables is randomly selected from all available variables;
- The best variable/split-point among them is chosen;
- The node is split into two nodes;
- The ensemble of trees is output;
- A prediction at a new point is made according to the regression or classification.

The accuracy of RF is comparable to that of AdaBoost, and RF is more robust to errors and outliers depending on the strength of the individual classifiers and the measure of dependency between them. The idea is to maintain the strength of the individual classifiers without increasing their correlation [35]. Overfitting is not a problem as the generalization error for a forest converges when the number of trees in the forest is large.

### Neural network

An NN is a set of connected input/output units in which a weight is associated with each connection [35]. For classification problems, the network fits the weights to predict the correct class label of the input tuples, such as the network topology or “structure”, empirically.

NN algorithms are inherently parallel. Parallelization techniques can be used to speed up the computation process. Several types of NN algorithms are available, and the most popular is backpropagation. This algorithm constructs a feed-forward multi-layered NN. A multi-layered NN consists of an input layer, one or more hidden layers, and an output layer [35].

Each of these layers is composed of units: the inputs correspond to the attributes that pass through the layer and are weighted and fed simultaneously to a second layer of neuron units, the hidden layer. The outputs of these hidden layer units can then be inserted into other hidden layers. The number of hidden layers is arbitrary, although in practice, only one hidden layer is used. A nonlinear activation function is applied to the weighted outputs of the last hidden layer, and then, the results of this step are inserted as input layers in the units of the output layer, which issues the network forecast for the provided tuples [35].

The complete set of weights is denoted by *θ*. The set consists of the following [34]: *α*_0*m*_, *α*_*m*_, *m* = 1, 2, …, *M*, *M*(*p* + 1) and *β*_0*k*_, *β*_*k*_, *k* = 1, 2, …, *K*, *K*(*M* + 1)

For classification, either the quadratic error or cross entropy is used, as follows:

$$\begin{array}{c} R\left(\theta \right)=-\sum_{i=1}^{N}\sum_{k=1}^{K}y_{ik}logf_{k}\left(x_{i}\right) \end{array}$$ (5)

With the corresponding classifier *G*(*x*) = *argmax*_*k*_ *f*_*k*_(*x*). with the softmax activation function and the cross entropy error function, the NN model is a logistic regression model in the hidden layers, and all parameters can be estimated by the maximum likelihood method. [34]. The softmax activation function is as follows [34]:

$$\begin{array}{c} g_{k}\left(T\right)=\frac{e^{Tk}}{\sum_{k=1}^{T}e^{Tl}} \end{array}$$ (6)

Backpropagation learning is a process in which the prediction of each tuple is compared to the target value, which can be the class label. The starting values for weights are typically chosen to be random values near zero [34]. The weights are then modified to minimize the error between the prediction and the value observed in the backwards direction (from the output layer) through each hidden layer to the first hidden layer. In general, the weights converge, and the process is terminated [35].

Generally, the input variables in the training phase should be normalized to fall between 0 and 1, and the variables with discrete values with *k* categories are transformed into *k* variables assuming values 0 and 1 [35].

### Variables

The variables were collected from the TRF4 Business Intelligence System. Usually, the lawsuits in these data originated in the first instance, and can be tracked to the second instance, which is the focus of this work. Time in this paper refers to the time for which the lawsuit uses resources.

**Fig 1:**
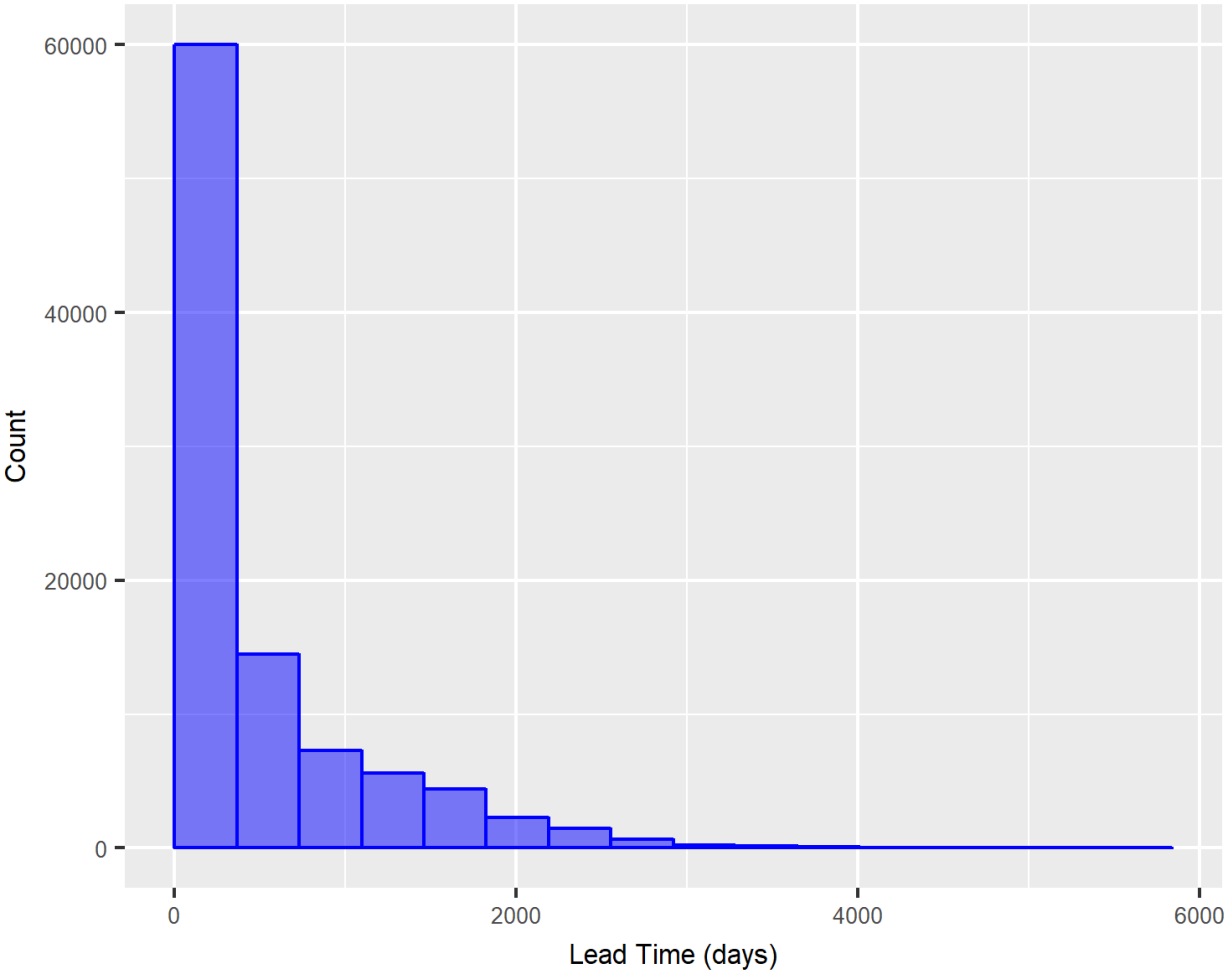
Histogram of lead time.

The dependent variable is the overall length of the case or the time between filing and the disposition of non-criminal lawsuits finished in 2016 in the 2^*nd*^ Instance Courts. This variable is known as lead time. This variable had an average of 498.3 days and a standard deviation of 611.63. The median was equal to 212.5 days, and thus, the lead time distribution is asymmetric positive. Fig 1 displays the histogram in 365 day intervals. The variable was grouped into bands with the objective of obtaining categories for prediction in years in such a way that the variable represents shorter, intermediate, long and extra-long durations, as follows:

- Up to 1 year, with 62.13% of observations;
- From 1 to 3 years, with 22.51% of observations;
- From 3 to 5 years, representing 10.34% of lead times;
- More than 5 years, or 5.02% of observations.

This paper addresses only the civil lawsuits regulated by the New Code of Brazilian Civil Procedure (CPC), which has been in force since March 2016, but not all the analyzed lawsuits in this study followed the new code. With the new CPC, the judgment must comply with the chronological order of completion to render a sentence or decision [43]. However, there are exceptions, such as repetitive processes, motions for clarification, internal appeals and urgent causes of judgment, which are recognized by a reasoned decision. According to Article 1048, there are also judging priorities for people 60 years of age or older or those suffering from severe illness [43].

The variables were chosen based on the literature and their availability. The lead time or overall case length was found through the literature review [7] and is available in the database. For different subsections of the literature, the covariates found in the literature review were grouped by characteristics of the action, the profile of the defendant and plaintiff, characteristics of the court and phases of lawsuit. The variables class [32], subject and type (physical or electronic) can be classified as characteristics of the action. The entity variable represents the defendant and plaintiff profile, and the characteristics of the court are described by the lawsuit variables Cabinet and Origin [32]. Other important variables reported in the literature review, such as the number of lawyers [32], legal complexity [10], total number of defendants, and total number of plaintiffs [26], were not available from Business Intelligence (BI) and were excluded from collection and analysis, although they are considered important for predicting the lead time. In future works, these variables could be collect by crawlers on the TRF4 website or from its database.

The independent variables are categorical and were transformed into binary variables according to their categories, resulting in the following variables:

- Cabinet of Appellate Justice (18 variables, 6 on each of the following issues: tributary, administrative and social security)
- Class, with the most frequent classes chosen being the following: civil appeal, interlocutory appeal instrument, necessary appeal/re-examination, necessary civil re-examination, motion to reverse or annul, writ of mandamus mandado judicial (class), termination, and termination (section), resulting in 8 classes;
- Origin of the process (three variables, namely, JF if the lawsuit is of recursal degree coming from the Federal Justice, JE if the lawsuit is from locations where there is no Federal Justice and TRF when the origin is the court itself);
- Electronic lawsuit. The variable assumes a value of 1 if the lawsuit is virtual and a value of 0 if the lawsuit is physical;
- Institution (the institutions that are parties of the lawsuit: INSS, Fazenda Nacional, Caixa Econômica Federal, Advocacia Geral da União, Ministério Público Federal, Estado—Paraná, Rio Grande do Sul ou Santa Catarina -, Município, Universidade, DNIT, Conselhos Regionais e Federais, Bancos, IBAMA, FNDE, EMGEA, União Federal, IFES, ANTT, Eletrobrás, INMETRO, and INCRA). These represent the Federal Union itself, social security offices, federal banks, state offices, federal councils, federal offices related to environment, economic development, energy, patents and property rights, transportation, among others;
- Defendant. If the parties cited in the Institution variable are defendants in the lawsuit;
- Plaintiff, if the parties listed in the Institution variable are plaintiffs in the lawsuit. There can be more than one institution, and thus, the same institution can be a defendant and plaintiff at the same time;
- Subject: the main issues, comprising the 96 subjects with the highest frequency;
- Lead time: The time in days between the filing and disposition in the Court of 2^*nd*^ Instance.

Lawsuits were categorized using the Table of Classes of Federal Justice. The classes identify the procedure indicated in the initial petition and aim to standardize the terminology among the organs of the judiciary. Not all classes were used; rather, only the most common classes were used. As with the classes, the lawsuit subject was categorized using the Table of Issues of Federal Justice.

The Federal Justice addresses conflicts between citizens and the Federal Public Administration. Thus, on one side of the conflict, there are public companies such as the INSS, federal public autarchies and foundation such as IBAMA, the councils of supervision and the Union.

**Fig 2:**
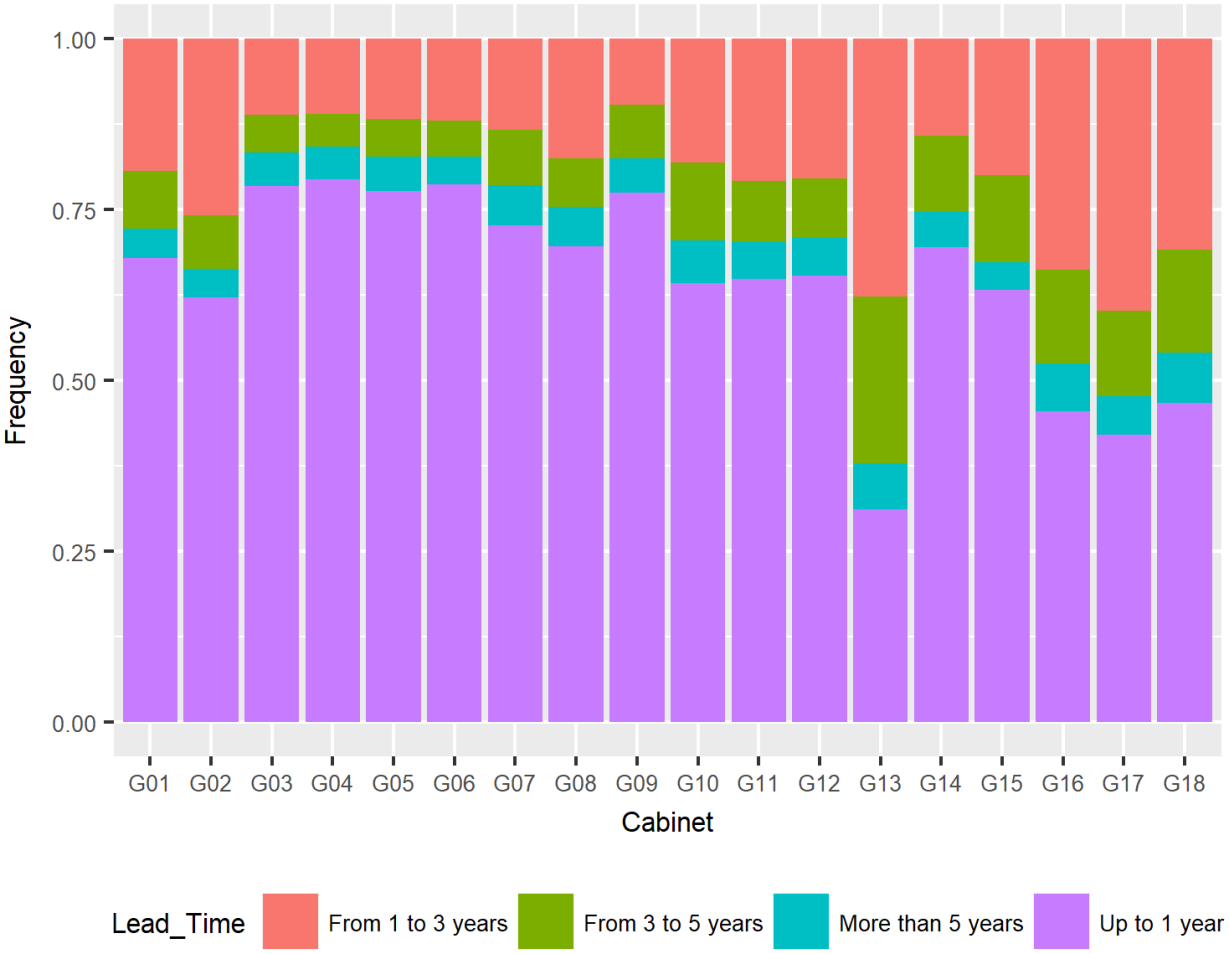
Distribution of lead time by cabinet.

Next, the lead time graphs are presented by categorical variable. Fig 2 shows the lead time according to the Cabinet of Justice. Cabinets G01 to G06 are tributary, G07 to G12 are administrative and G13 to G18 are social security, and all showed a lower percentage of lawsuits classified in the category of up to one year, especially Cabinet G13 (31.17%).

**Fig 3:**
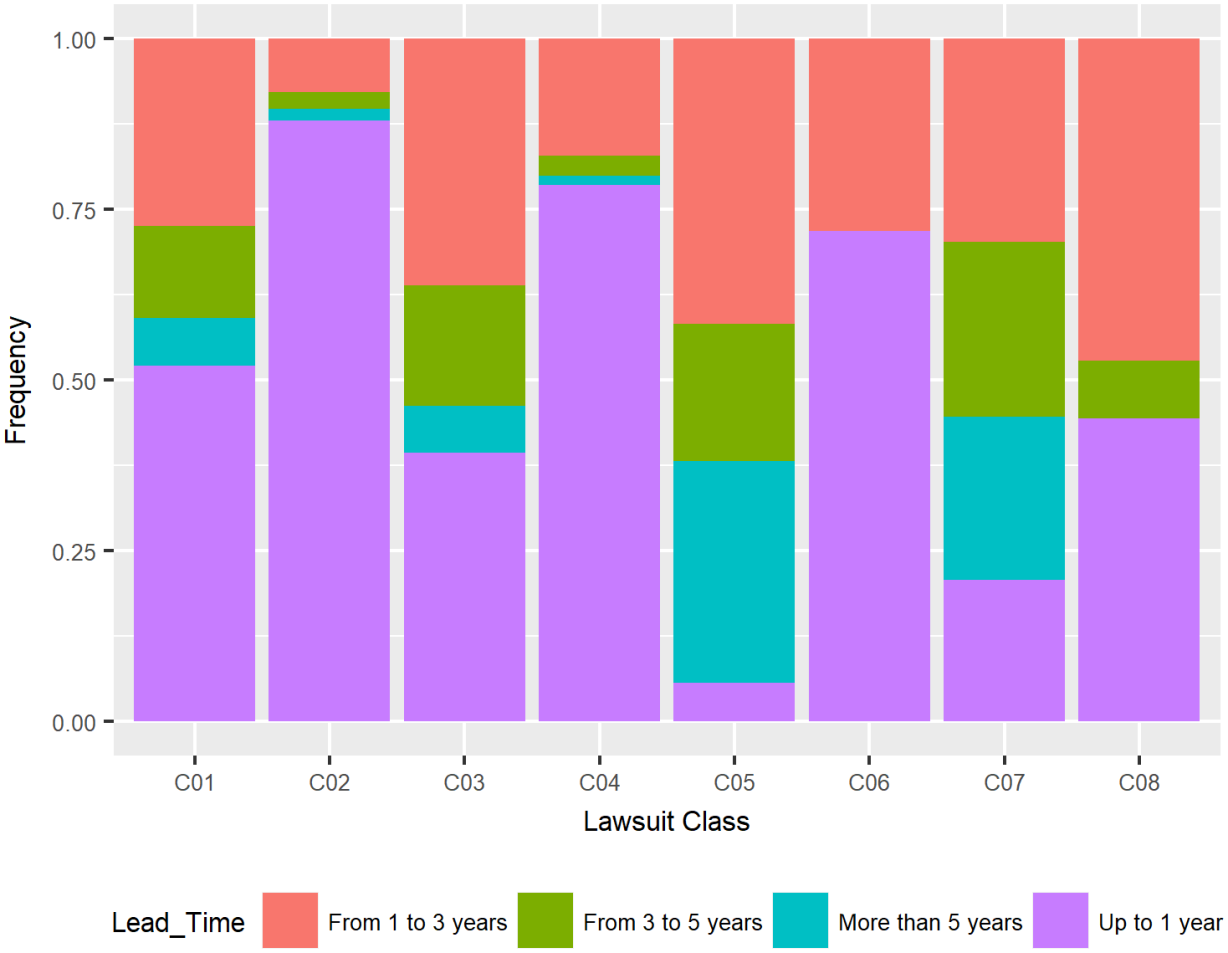
Distribution of lead time by lawsuit class.

Classes C05, C03 and C07 presented a lower percentage of lawsuits with lead times of up to one year: for lawsuits of process class C03, 39.37% had times of up to 1 year, 5.60% of the processes of class C05 had times of up to one year and 20.61% of the processes of class C07 had times in this category, according to Fig 3.

**Fig 4:**
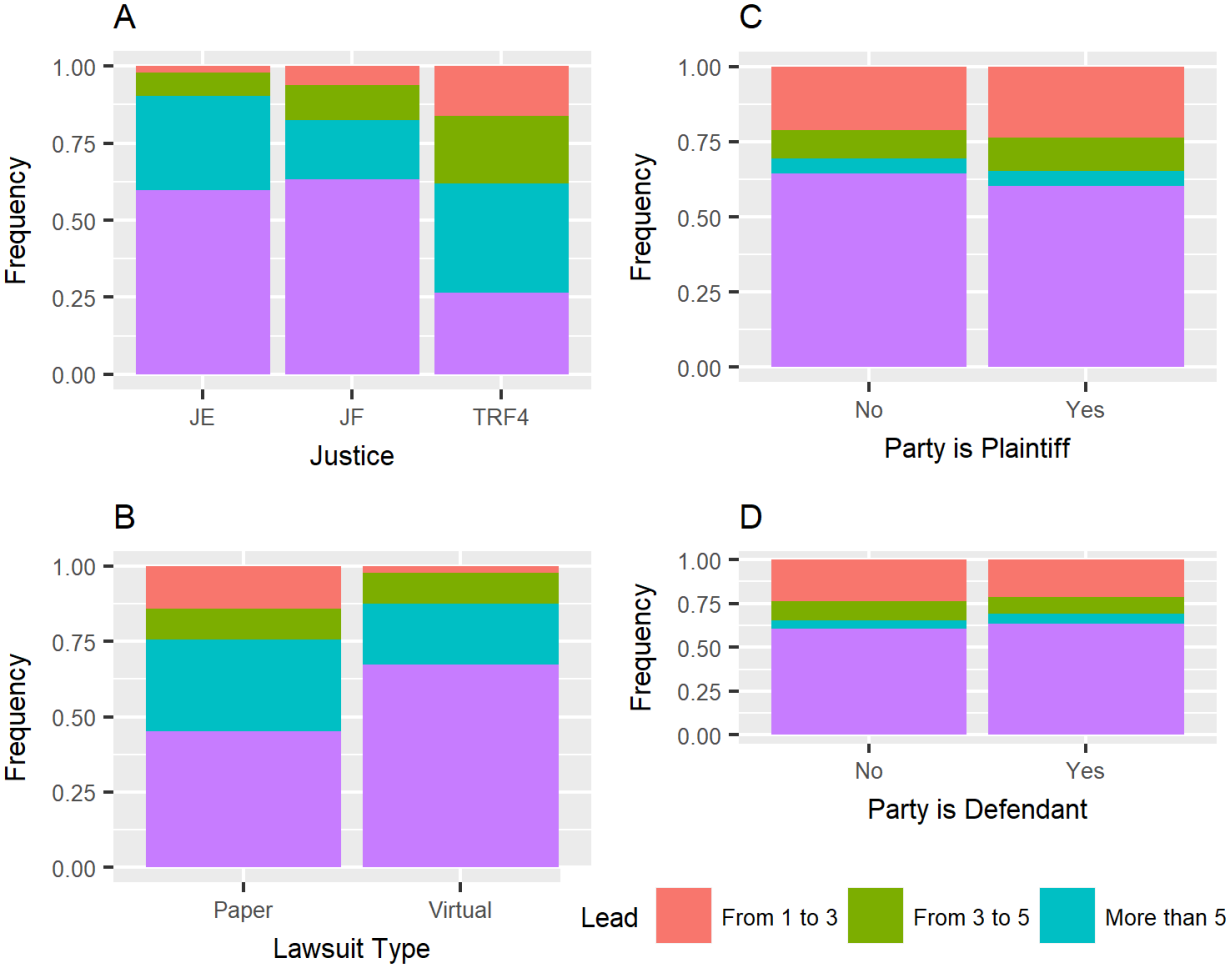
A—Justice type and lead time, B—lawsuit type and lead time, C—main plaintiff and lead time, D—main defendant and lead time.

The Federal Justice and Justice of State lawsuit classes had lead times of up to one year, 63.21% and 59.77% respectively, while the origin of the TRF case had a one-year lead time of 26.56%, according to Fig 4.

Electronic lawsuits had more lead times falling in the category of up to 1 year than physical processes: 67.41% of electronic lawsuits had a lead time of up to 1 year, while 45.18% of physical lawsuits had such lead times.

**Fig 5:**
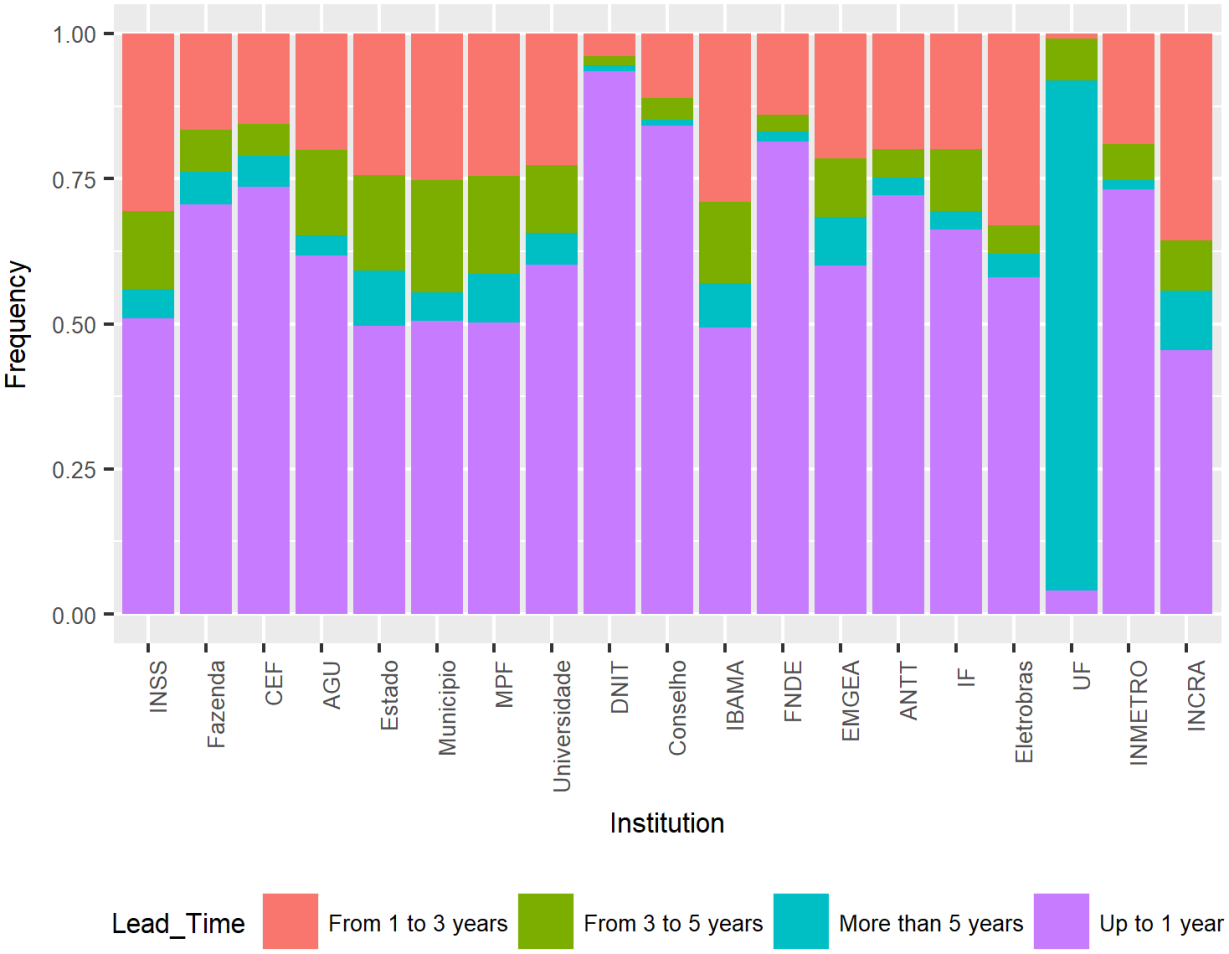
Distribution of lead time by party.

The institutions with the highest frequency of occurrence were selected for analysis. Among these parties, according to Fig 5, the União Federal (UF) had the lowest percentage of lead time of up to 1 year: 4.13%, while 93.56% of the lawsuits of the DNIT institution had a lead time of up to 1 year. When comparing the time of defendants and plaintiffs (with the institutions shown in Fig 5), the lead time percentages of the defendant institutions were 63.62%, and those of the plaintiff institutions were 60.23% according to Fig 4.

At first, a linear regression model was constructed with the described variables in order to verify the error of the predictions and the quality of the adjustment, although the assumptions of the model were not verified. The mean square of the residuals, which is equal to 480.7, is close to the mean of the lead time, 498.3 days, and although 148 terms were added to the model, the adjusted R-squared, which is equal to 0.382, indicates a poor quality of fit.

The NB approach assumes independence between the explanatory variables. To prove this assumption, the average quadratic contingency coefficient was calculated. This coefficient, which is obtained by dividing the chi-square statistic by the data dimension, is not influenced by sample size and can hold a minimum value (0) but does not have a maximum value. The coefficient was equal to 0.92 for the Author and Defendant variables, indicating dependence between them. However, these variables were maintained in all models because the other approaches do not have this assumption.

A stated above, the NB approach assumes independence between the explanatory variables. In another approach to proving this assumption, a chi-square test with continuity correction among the independent variables was performed, resulting in 21756 comparisons. However, the assumptions for the test (a number of observations greater than 5 or a minor expected frequency greater than 5 [44]) were not checked. Then, the average quadratic contingency coefficient was calculated for all independent variables. This coefficient is obtained by dividing the chi-square statistic by the data dimension. This coefficient can hold a minimum value (0) but does not have a maximum value. For example, for the variables Author and Defendant, with the application of the chi-square test, the null hypothesis of independence is rejected (p value = 0.00). As the test is influenced by sample size, rejection of the null hypothesis is expected. In the case of these two variables, the coefficient was equal to 0.92, and the association can be considered strong (coefficient closer to 1.0 [45]). However, all variables were maintained in the models because the other approaches do not have the independence assumption.

### Model evaluations

In this study, the classification models are evaluated using measures of accuracy, precision, specificity, sensitivity, F1 and k-fold cross-validation.

To calculate the measures of accuracy, specificity, sensitivity, precision and F1, a confusion matrix is used in which items are classified into the positive and negative categories and then, the true positives, false positives, true negatives and false negatives are totaled. The meaning of the measures is as follows [42]:

- Accuracy is the quantity of items correctly classified in relation to the total number of items;
- The precision and sensitivity measurements are calculated together: precision is the ratio of the number of positive items correctly classified to the number of items classified as positive, and sensitivity is the fraction of positive items correctly detected by the classifier in relation to all positive items;
- Specificity is the fraction of negative items correctly detected by the classifier in relation to all negative items.

Because there are four time categories, the measures specificity, sensitivity, precision and F1 are calculated for each of the categories, and the accuracy, in addition to the calculation for each category, is calculated as a a global index.

The steps of the k-fold cross-validation method are as follows [35]:

- The initial data are randomly divided into *k* mutually exclusive subsets *D*_1_, … *D*_*k*_ with approximately the same number of items;
- In iteration i, *D*_*i*_ is reserved for the test, and the remaining partitions are used to train the model. Each partition is used the same number of times for training and once for testing;
- Usually, k = 10 is recommended because of the low bias and variance of this setting.

### Model fit

The variables were collected from BI from TRF4, resulting in a database with 99,773 records, of which 96,578 were complete. The independent variables are categorical and were transformed into binary according to their categories, resulting in 148 variables, while the dependent variable is the lead time, which was categorized in 4 bands as described in the variables subsection.

The dataset was divided in two subsets: 80% (77,262 lawsuits) of the dataset was used for training, and 20% (19,316) was used for testing. Before being split, the records were ordered randomly because they were ordered by type of lawsuit. After fitting the models in the training phase, each fitted model was applied to the test lawsuits, and then, the evaluation measures were calculated. Finally, k-fold validation was performed for the SVM, NB, RF and NN approaches.

To fit the models, R was used. The e1071 package was used to fit the SVM model. This package uses a one-to-one approach for classifying several categories in which k*(*k* − 1)/2 binary classifiers are trained, and the appropriate category is selected by a voting scheme. The cost and *γ* parameters were 0.95 and 0.3, respectively.

The radial kernel chosen for the training and predicting processes was recommended in the literature [39] describing the steps for an SVM analysis in the subsection on SVM. To obtain the gamma and cost parameters, twenty SVM models with combinations of costs and gammas values were tested in relation to total accuracy. Models with greater values of this measure were chosen.

To fit the NB model, the e1071 package was used again. The algorithm used in this package computes the a posteriori conditional probabilities of a categorical (dependent) variable with independent predictor variables using the Bayes rule [46].

To fit the RF model, the randomForest package was used. The package implements the Breiman algorithm, which is based on the original Fortran code of Breiman and Cutler for classification and regression [47].

As in the choice of SVM parameters, twenty models with combinations of numbers of trees and numbers of variables were tested. The models with greater total accuracy were chosen. Although the literature recommendation for the number of variables, *p*, was $\sqrt{p}$ in the classifications and $\frac{p}{3}$ in the regression [34], the chosen number was 50. For the number of trees to grow, the recommendation is not to set a number that is too small to ensure that every input row gets predicted at least a few times [47]. The number of trees was 500.

For the other model parameters, node size and cut off, the default values in the randomForest package were chosen. As the algorithm uses a bootstrap sample, a random seed was fixed for repeatability.

For NNs, the nnet package was used. This package is used for feed-forward NNs with a single hidden layer and for multinomial log-linear models [48]. The parameters were chosen according to the literature review and the recommendations in the nnet package specifications. As the number of units in the hidden layers is arbitrary and there are no clear rules for finding the best number of hidden layers, the design is a trial and error process [35].

Accuracy can be affected by the initial weights and the number of hidden layers. Thus, when the accuracy of a trained network is considered unsatisfactory, the training process is repeated with different topologies and initial weights [35].

Using the trial and error process, the number of hidden layers was set equal to 5. For the initial weights, the recommendation in the literature review was to choose random values near zero [34]. Thus, values within the interval [−0.1; 0.1] were chosen.

These values were tested in the posterior evaluation phase by cross-validation techniques, as described in the subsection on model evaluation. The maximum number of iterations was equal to 500. After evaluating the models, the values were considered acceptable. The values are in presented in Table 2.

**Table 2:** Model parameters.

Approach	Parameters
Support Vector Machine	Function = Kernel, Gamma = 0.95, cost = 0.3
Naive Bayes	No parameters
Random Forest	Number of variables = 50, number of trees = 500
Neural Network	Hidden layers = 5, Initial weights in [-0.1, 0.1], decay = 0.0005, maximum number of iterations = 500

Source: The authors.

The next section presents the results of the fit of the four models, the model comparisons, the evaluation of these models according to the variables, and the proposed evaluation and fits described in this section.

## Results and discussion

Table 3 presents the results of the application of the models according to the measures accuracy, sensitivity, specificity, precision and F1. For the comparison, a confusion matrix was created for each of the classes by lead time category.

**Table 3:** Measures of model performance in the test phase.

Approach and Class	Accuracy	Sensitivity	Specificity	Precision	F1
**Up to 1 year**					
Support Vector Machine	77.58	91.61	54.41	76.84	83.58
Naive Bayes	69.83	74.26	62.52	76.59	75.41
Random Forests	77.65	89.72	57.73	77.80	83.33
Neural Network	75.76	90.46	51.50	75.49	82.30
**From 1 to 3 years**					
Support Vector Machine	78.79	42.85	89.35	54.18	47.86
Naive Bayes	71.17	47.07	78.25	38.87	42.58
Random Forests	78.29	43.67	88.47	52.67	47.75
Neural Network	76.20	40.67	86.64	47.22	43.70
**From 3 to 5 years**					
Support Vector Machine	89.65	22.23	97.36	49.11	30.60
Naive Bayes	85.70	29.64	92.12	30.09	29.86
Random Forests	89.00	26.31	96.17	44.05	32.94
Neural Network	89.42	12.55	98.22	44.62	19.59
**More than 5 years**					
Support Vector Machine	97.67	58.49	99.62	88.43	70.39
Naive Bayes	96.72	36.39	99.72	86.49	51.23
Random Forests	97.46	57.05	99.47	84.19	68.01
Neural Net	97.60	55.52	99.70	90.07	68.70

Source: The authors.

The higher the value of these measures is, the better the prediction of model is. The values for the SVM and RF approaches were close for all measurements and lead time categories. The values of the measures for the NB approach were lower than those of the other approaches, indicating a worse performance. The accuracy was not high, mainly for the lead time category of up to 1 year. For the SVM model, the accuracy was 77.58%. For the model using NB, the accuracy was 69.83%. For the model using RF, the accuracy was 77.65%, and for NN, the accuracy was 75.76%. The specificity, the measure of correctly classified negatives in relation to the total number of negatives, was lower for the category of lead times of up to 1 year in comparison to the other categories for all models. For the NN model, the specificity in the category of up to 1 year was 51.50%, the lowest.

The sensitivity and precision measures, which measure the correct classification of positive items, were higher for the category of lead time up to one year and lower for the category of 3 to 5 years for all models. The F1 measure, the mean of the confirmation and utility calculated through the harmonic mean of the sensitivity and precision [42], for the SVM model for the category of up to one year was 83.58% and for the category from 3 to 5 years was 30.60%.

That is, the correct evaluation of positives was greater for the category of up to 1 year and smaller for the others, while the evaluation of negatives was greater for the other categories of lead time. This result was expected because the category of up to 1 year contained the most entries: 62.28% of test data fell in this category of lead time. The category of 3 to 5 years, which exhibited the lowest values of the measures referring to the classification of positive precision and sensitivity, represented 10.27% of the test data. When considering all categories, the total accuracy of the model was, 71.84, 61.71, 71.20 and 69.49%, for SVM, NB, RF and NN, respectively, according to Table 4.

**Table 4:** Accuracy of the models in the test phase and the 10-fold cross-validation.

Approach	Test phase	10-fold cross-validation (mean ± standard deviation)
Support Vector Machine	71.84	71.47 ± 0.55
Naive Bayes	61.71	60.64 ± 0.60
Random Forests	70.79	70.85 ± 0.52
Neural Network	69.49	69.78 ± 0.48

Source: The authors.

For the evaluation of the models, k-fold cross-validation was used. The dataset was randomly ordered, divided into 10 approximately equal parts, and then, the steps proposed by [35] were followed. In the evaluation phase, the performance of the models was similar to that of the test phase for all measures and lead time categories, indicating that although the values of the measurements are not high, they are constant, which can be observed by the standard deviation of 10 repetitions as shown in Tables 4 and 5.

**Table 5:** Measures of performance of the models in the test phase and the 10-fold cross-validation (mean ± standard deviation).

Approach	Accuracy	Sensitivity	Specificity	Precision
**Up to 1 year**				
Support Vector Machine	77.39 ± 0.45	91.17 ± 0.38	54.78 ± 0.98	76.78 ± 0.59
Naive Bayes	68,70 ± 0,44	73,29 ± 0,46	61,16 ± 0,95	75,58 ± 0,55
Random Forests	77.40 ± 0.36	89.49 ± 0.39	57.56 ± 0.90	77.57 ± 0.51
Neural Network	75.79 ± 0.49	91.83 ± 0.80	49.48 ± 2.58	74.90 ± 0.78
**From 1 to 3 years**				
Support Vector Machine	78.62 ± 0.40	42.14 ± 1.29	89.22 ± 0.28	53.17 ± 0.74
Naive Bayes	70.53 ± 0.59	46.23 ± 1.11	77.60 ± 0.55	37.48 ± 0.87
Random Forests	78.10 ± 0.42	42.06 ± 1.08	88.57 ± 0.32	51.67 ± 0.91
Neural Network	77.25 ± 0.50	37.19 ± 2.46	88.89 ± 0.96	49.34 ± 1.05
**From 3 to 5 years**				
Support Vector Machine	89.55 ± 0.35	24.74 ± 1.78	97.02 ± 0.21	48.98 ± 3.02
Naive Bayes	85.65 ± 0.38	29.52 ± 1.32	92.12 ± 0.44	30.21 ± 1.85
Random Forests	88.85 ± 0.35	28.43 ± 2.30	95.81 ± 0.25	43.90 ± 2.59
Neural Network	89.14 ± 0.29	15.77 ± 3.60	97.59 ± 0.44	42.74 ± 4.20
**More than 5 years**				
Support Vector Machine	97.38 ± 0.16	55.43 ± 1.76	99.60 ± 0.09	88.07 ± 2.63
Naive Bayes	96.39 ± 0.19	32.73 ± 1.57	99.76 ± 0.05	87.88 ± 2.44
Random Forests	97.24 ± 0.17	55.48 ± 1.59	99.44 ± 0.08	84.20 ± 1.82
Neural Network	97.37 ± 0.15	54.20 ± 2.12	99.65 ± 0.09	89.44 ± 2.47

Source: The authors.

For comparing the approaches, univariate analyses of variance were conducted considering as dependent variables: the accuracy by lead time category; the overall accuracy; the specificity; and the F1 measure. A randomized blocks experimental design was used where each block is the lead time category with 10 replications (k-folds). For the analysis of total accuracy, the experiment was completely randomized. The treatments for all cases were the analysis methods (SVM, NB, RF and NN). After the analysis and verification of the assumptions of the analysis of variance, Tukey’s multiple comparisons test was performed to verify the differences among the treatments.

All analyses of variance were significant (p value ≤ 0.01), indicating evidence that at least one of the four approaches is different in relation to the assessment measure. Regarding the conclusion of the analysis of multiple comparisons of accuracy, according to the lead time category, there is evidence of significant differences (p value ≤ 0.04) among all pairs of means with the exception of RF and NN (p value = 0.37) and between RF and SVM (p value = 0.70). Regarding specificity, there is evidence of significant differences between the SVM and NB approaches and between RF and NB, both with p values of ≤ 0.00.

The F1 measure, which measures jointly the precision and sensitivity, indicates is that there is evidence of differences among all approaches in pairwise comparisons except for between RF and SVM (a p value ≥ 0.99).

The multiple comparison test for the total global accuracy shows that there is evidence of differences across all approaches (p value ≤ 0.05 for all pairwise comparisons). The multiple comparisons with blocks or among lead time categories were significant for all pairs of approaches (p value ≤ 0.00) for both accuracy, specificity and F1.

The performance of the SVM and RF approaches are very close for all measures, and there is no evidence of differences, although the values for SVM are higher than those for RF except for the F1 for the lead time category of 3 to 5 years. The SVM F1 was 30.60, and that for RF was 32.94%. The NB approach produced the lowest values for these measurements, as shown in Table 4.

The observations are about the model variables. The variables available in BI are categorical and available when the lawsuit is filed in the 2^*nd*^ Instance Court. With the addition of other variables that are not available in the database, the model would likely present higher values for the evaluation measures.

According to the results of the analysis of variance and the test of multiple comparisons, in Table 6, the SVM classifier presented the best results for total accuracy, accuracy by class and F1. Although the SVM classifier does not have the best result for specificity, which indicates type 2 error, there were no significant differences between this classifier and RF (p value ≥ 0.99) and NN (p value ≥ 0.36) according to the multiple comparisons test. Given the results, and according to the measures evaluated for the specific case, the SVM can be considered the best classifier.

**Table 6:** Comparison between the approaches according to the accuracy, specificity and F1 in the evaluation phase—results of Tukey’s multiple comparisons test.

Measure	Best performance
Class accuracy	SVM* and RF (p value ≥ 0.70)
Specificity	RF*, SVM and NN (p values ≥ 0.99, ≥ 0.23 and ≥ 0.36, respectively to RF-SVM, RF-NN and SVM-RF)
F1	SVM* and RF (p value ≥ 0.99)
Total accuracy	SVM (p value ≤ 0.05)

Source: The authors.

* greater value

The variable response, lead time, which was initially measured in discrete days, was transformed into a categorical variable to obtain a prediction lead time class. This categorization resulted in unbalanced categories: the first category, with a time equal to or less than 1 year, has 62.09% of the lawsuits in the training phase and 62.28% of those in the test phase. In this way, the evaluation measures were different among the categories, and the category of 3 to 5 years had the lowest values of accuracy for all models.

## Conclusion

The prediction of lawsuit lead time is an incipient theme in the literature, with data mining and regression analysis being the most commonly used approaches. The main objective of this article was to contribute to the literature on models with lawsuit lead time as a categorical variable, a theme that has been little explored in the academy. The model presented here can be used for the prediction of the class of lead time when a lawsuit is registered in the 2^*nd*^ Instance Courts.

The predictive variables used here are categorical and are available in TRF4 BI. The dependent variable, initially measured in days, was recoded in 4 categories according to the number of years. In the specific case analyzed, to obtain a better accuracy, other variables, such as the number of lawyers and parties, could be included.

Regarding the approaches used for the comparison, SVM obtained the best evaluation measures, followed by RF, although there were not significant differences between these approaches. The fit of the four approaches was verified through k-fold cross-validations, resulting in evaluation measures that were very close to the fit of the models. If other parameters, such as the number of hidden layers, had been used for the NNs, the outcomes of this approach may could have been improved.

Both the SVM and RF approaches can be used for the prediction of the lead time class of civil lawsuits, although the performance measures were low. To improve these performance measures, others approaches to class prediction, such as AdaBoosting, Bayesian networks and logistic regression, could be used.

For future studies, the inclusion of other predictor independent variables is suggested, as well as an analysis not only of the 2^*nd*^ Instance Courts but of the whole lifetime of a lawsuit from entrance in the 1st Instance Court to the end. Regarding analysis techniques, the use of hybrid methods is suggested. In addition, analysis with quantitative response variables while introducing other variables in the study or using another technique such as survival analysis may be avenues for future study.

Using text mining, which is the process of analyzing text looking for patterns, may also be important for extracting useful information [49]. In this sense, complex networks (CNs) may be useful as these approaches may be able to capture syntactic, semantic and even pragmatic features in text classification [13]; modeling of texts as CNs [15]; devising complex networks methods of text classification by a fuzzy classification strategy [12]; analyzing the spatial distribution of a word [14]; and in sentiment analysis [16].

The processing time for each of the approaches was not controlled in this work. For future works, it is suggested that this time be included as a variable along with the total accuracy measure for choosing the model parameters. In addition, the processing time can be a variable response for the comparison of approaches, along with measures of total accuracy, accuracy by class, F1 and specificity.

There is a debate as to whether jurimetrics can contribute to achieving the desired reasonable duration of a lawsuit. However, jurimetrics can be used as an important auxiliary tool in such an analysis based on the need for compatibility between the flow and process management and judicial policy (strategic planning, organization and management of the judiciary) [24]. Jurimetrics, or quantitative methods applied to law, are believed to be able to contribute to the reduction of lead time because these approaches provide knowledge about lawsuit duration.

## Supporting information

S1 File Database file. Cleansed raw data used in the study (.txt).
(ZIP)
